# Stimulus-Induced Activation of the Glycoprotein Hormone α-Subunit Promoter in Human Placental Choriocarcinoma Cells: Major Role of a tandem cAMP Response Element

**DOI:** 10.3390/cimb46040202

**Published:** 2024-04-07

**Authors:** Lars Bürvenich, Oliver G. Rössler, Gerald Thiel

**Affiliations:** Department of Medical Biochemistry and Molecular Biology, Saarland University, Building 44, 66421 Homburg, Germany; l-buervenich@web.de (L.B.); oliver.roessler@uks.eu (O.G.R.)

**Keywords:** CREB, designer receptor, forskolin, MEKK1, MKK6, PKA, glycoprotein hormone α-subunit

## Abstract

The glycoprotein hormones LH, FSH, TSH and chorionic gonadotropin consist of a common α-subunit and a hormone-specific β-subunit. The α-subunit is expressed in the pituitary and the placental cells, and its expression is regulated by extracellular signal molecules. Much is known about the regulation of the α-subunit gene in the pituitary, but few studies have addressed the regulation of this gene in trophoblasts. The aim of this study was to characterize the molecular mechanism of stimulus-induced α-subunit gene transcription in JEG-3 cells, a cellular model for human trophoblasts, using chromatin-embedded reporter genes under the control of the α-subunit promoter. The results show that increasing the concentration of the second messengers cAMP or Ca^2+^, or expressing the catalytic subunit of cAMP-dependent protein kinase in the nucleus activated the α-subunit promoter. Similarly, the stimulation of p38 protein kinase activated the α-subunit promoter, linking α-subunit expression to stress response. The stimulation of a Gαq-coupled designer receptor activated the α-subunit promoter, involving the transcription factor CREB, linking α-subunit expression to hormonal stimulation and an increase in intracellular Ca^2+^. Deletion mutagenesis underscores the importance of a tandem cAMP response element within the glycoprotein hormone α-subunit promoter, which acts as a point of convergence for a multiple signaling pathway.

## 1. Introduction

The glycoprotein hormone chorionic gonadotropin (hCG) can be detected in the serum of pregnant women 6 to 9 days after conception. The level continues to increase during pregnancy and peaks about 8 to 10 weeks post-conception. hGC maintains the synthesis and secretion of progesterone during early gestation by the corpus luteum of the ovaries and later by the placenta and is crucial in early pregnancy [1].

The hormone consists of two non-covalently linked subunits, the α-subunit and the β-subunit, and shares structural homology with the pituitary hormones: luteinizing hormone (LH), follicle-stimulating hormone (FSH), and thyroid-stimulating hormone (TSH). In fact, all four glycoprotein hormones share a common α-subunit, whereas the β-subunits are distinct and are responsible for the biological specificity of each glycoprotein hormone. The glycoprotein hormones play a crucial role in the regulation of reproduction, growth, metabolism and pregnancy, and it is therefore an important topic in molecular endocrinology to decipher the regulation of the genes encoding the α- and β-subunits. Given the importance of hCG in early pregnancy, it is of great importance to elucidate the endocrine signaling cascades that regulate the expression of the α- and β-subunits of GC in placental cells.

Genetic control elements responsible for the tissue-specific expression of the glycoprotein hormone α-subunit have been identified in the proximal promoter of the α-subunit gene [2,3] and the glycoprotein hormone α-subunit promoter has been successfully used for transgene expression in the pituitary [4,5,6]. In addition, glycoprotein hormone α-subunit expression has been reported to be regulated by extracellular signaling molecules such as steroid hormones and ligands of G protein-coupled receptors and receptor tyrosine kinases [2,3].

Stimulus-induced regulation of the promoter of the glycoprotein hormone α-subunit has mainly been studied in pituitary cell lines [7,8,9,10,11,12,13,14,15,16,17]. Only a few studies addressed the regulation of this gene by extracellular and intracellular stimuli in trophoblasts. It has been shown that stimulation of JEG-3 cells with pituitary adenylate cyclase-activating protein (PACAP) activates the promoter of the glycoprotein hormone α-subunit gene [18]. Similarly, the stimulation of trophoblast cell lines with epidermal growth factor has been shown to activate a reporter gene under the control of the human glycoprotein hormone α-subunit promoter [19,20]. In addition, steroid hormones are involved in the regulation of the gene encoding the glycoprotein hormone α-subunit [21,22,23]. The purpose of this study was to elucidate the molecular mechanism of stimulus-induced glycoprotein hormone α-subunit gene transcription in JEG-3 cells, a cellular model system for human trophoblasts. We hypothesized that other than the cAMP signaling pathway would have an impact on the expression of the glycoprotein hormone α-subunit gene in trophoblasts. Experiments with immortalized pituitary cell lines indicated an involvement of the stress-activated protein kinases c-Jun *N*-terminal protein kinase (JNK) and p38 protein kinase [24,25]. In addition, a regulation of the expression of the glycoprotein hormone α-subunit by Gαq-coupled receptors, in particular by gonadotropin releasing hormone (GnRH) receptors, has been postulated [9,10,15,16].

We used lentiviral gene transfer to integrate a glycoprotein hormone α-subunit promoter-controlled reporter gene into the genome of JEG-3 cells. Previous reports addressing the regulation of the glycoprotein hormone α-subunit promoter were performed with transient transfection experiments of plasmids. However, transfected plasmids are not or only partially organized into nucleosomes, a typical feature of eukaryotic genes [26,27]. The integration of the reporter genes into the chromatin of the cells ensured that the reporter genes were embedded into a nucleosomal context identical to the structure of all other genes in JEG-3 cells. In addition, we have developed a reporter gene with a deletion of the tandem CRE within the glycoprotein hormone α-subunit promoter, allowing us to study the effects of this genetic element on stimulus-induced activation of the glycoprotein hormone α-subunit promoter. We hypothesized that the tandem CRE might represent a genetic landmark for different signaling pathways. For example, it has been shown that the tandem CRE is responsible for transcriptional activation of the glycoprotein hormone α-subunit promoter by epidermal growth factor [19]. The tandem CRE within the promoter of the glycoprotein hormone α-subunit gene functions as a binding site for the cAMP response element binding protein (CREB) [25] and may also bind other basic region leucine zipper proteins such as ATF2. CREB is a transcription factor that integrates numerous intracellular signaling cascades, particularly those leading to increased intracellular cAMP concentrations. In addition, CREB is activated after stimulation of Gαq-coupled designer receptors in HEK293 cells [28] and after stimulation of GnRH receptors in αT3-1 gonadotrophs [29].

## 2. Materials and Methods

### 2.1. Cell Culture and Reagents

Human JEG-3 placental choriocarcinoma cells derived from a human gestational choriocarcinoma were purchased from the German Collection of Microorganisms and Cell Cultures (DSMZ), Braunschweig, Germany. The cells exhibit many biological characteristics of early trophoblasts. They express and secrete large amounts of the glycoprotein hormone chorionic gonadotropin (hCG) [30,31]. Cells were cultured in DMEM medium containing 10% fetal calf serum, 100 units/mL penicillin and 100 μg/mL streptomycin. Stimulation with forskolin (20 μM, Calbiochem, Darmstadt, Germany, Cat # 344270, dissolved in DMSO) was performed for 24 h in a medium containing 0.05% fetal bovine serum. Forskolin is a diterpene isolated from *Coleus barbatus* that stimulates adenylate cyclase, triggering an increase in intracellular cAMP concentration. JEG-3 cells expressing Rαq-coupled designer receptors were incubated in DMEM containing 0.05% fetal bovine serum for 24 h before stimulation with clozapine-*N*-oxide (CNO) (1 μM, PubChem CID: 135445691, Enzo Life Sciences, Lörrach, Germany, #NS-105-0005) in a medium containing 0.05% fetal bovine serum.

### 2.2. Lentiviral Gene Transfer

We used lentiviral gene tranfer to express stimulus-responsive protein kinases or a dominant-negative mutant of the transcription factor CREB. The transgenes were expressed under the control of the human ubiquitin C promoter, in contrast to other reports that based their conclusions on transient transfection experiments with extremely high levels of plasmids [14,15,20]. The lentiviral transfer vectors pFUW-Rαq, pFUW-MKK6E, pFUW-MEKK1Δ, and pFUW-REST/CREB have been described elsewhere [28,29,32]. To generate the lentiviral vector pFUW-FLAG-NLSCα, encoding the catalytic subunit of protein kinase A together with a nuclear localization signal (NLS) derived from the SV40 large T antigen, we digested plasmid pCMV-FLAG-NLSCα [33] with Ecl136II and SmaI and cloned the fragment into HpaI cut pFUW. Viral particles were generated in HEK293-TN cells and the infections were performed as previously described [34]. In brief, the cells were seeded together with the virus, and incubated overnight. The medium was removed, and the cells were incubated for 24 h in DMEM medium. Then, the cells were incubated in DMEM medium containing 0.05% fetal bovine serum for 24 h. Stimulation was performed for 24 h in DMEM medium containing 0.05% fetal bovine serum. The number of seeded cells was empirically determined so that the cells were not confluent at the end of the incubation period.

### 2.3. Reporter Gene Assay

The lentiviral transfer vector pFWαGSU.luc was generated by inserting the promoter of the human glycoprotein hormone α-subunit gene (sequence −846 to +48) from plasmid −846αLUC [9] into a lentiviral transfer vector upstream of the luciferase open reading frame. Plasmid −846αLUC was a kind gift of Jacky M. Burrin, St. Bartholomew’s and London School of Medicine and Dentistry. To construct the plasmid pFWαGSUΔCRE.luc, containing a deletion of the tandem CRE sequence, we first subcloned the αGSU promoter into pBluescript (Stratagene, La Jolla, CA, USA). The newly generated plasmid pBSαGSUprom was digested with AatII and then incubated with T4 DNA polymerase (New England Biolabs Inc., Frankfurt, Germany; 1 unit polymerase/μg plasmid) for 15 min at 12 °C in the presence of bovine serum albumin (final concentration 100 μg/mL) according to the manufacturer’s instruction and as described [35]. The T4 DNA polymerase was inactivated using incubation for 20 min at 75 °C in the presence of EDTA (final concentration 10 mM). The plasmid was ligated with T4 ligase and transformed into bacteria. Cell extracts were prepared using reporter lysis buffer (Promega, Cat. No E4030, Mannheim, Germany). Luciferase activities of the extracts were measured using a luminometer (Berthold Detection Systems, Huntsville, AL, USA). The light units were normalized to the protein concentration of the extracts, determined using a BCA protein assay kit (Thermo Scientific^TM^, Cat. No. 10678484).

### 2.4. Western Blots

FLAG-tagged NLS-Cα protein was detected in Western blot experiments using M2 monoclonal antibody (Sigma-Aldrich, Steinheim, Germany, #F1804) at a dilution of 1:3000 in TBS. A total of 20 µg of nuclear proteins were separated on a 10% SDS-PAGE. Wet transfer was performed for 5 h at 200 mA. The membrane was blocked for 45 min with slim fast powder (4%) in TBST buffer. A peroxidase-conjugated goat-anti-mouse antibody (Jackson ImmunoResearch Laboratories, Inc./Dianova, Hamburg, Germany (#115-035-003)) was used as the secondary antibody at a 1:20,000 dilution with an incubation time of 3 h. Immunoreactive bands were detected with enhanced chemiluminescence using a 1:1 solution of solution 1 (100 mM Tris-HCl, pH 8.5, 5.4 mM H_2_O_2_) and solution 2 (2.5 mM Luminol, 400 μM p-coumaric acid, 100 mM Tris-HCl, pH 8.5). Chemiluminescence detection was performed using a ChemiDoc XRS+ imaging system from Bio-Rad (Bio-Rad Laboratories GmbH, München, Germany).

### 2.5. Statistics

The two-tailed Student’s *t*-test was used for the statistical analyses. The statistical probability is expressed as *** *p* < 0.001; ** *p* < 0.01, and * *p* < 0.05. We considered the values significant when *p* < 0.05.

## 3. Results

### 3.1. Forskolin Activated Gene Transcription of a Chromatin-Integrated Human Glycoprotein Hormone α-Subunit Promoter-Controlled Reporter Gene in JEG-3 Human Placental Choriocarcinoma Cells

The stimulation of αT3-1 gonadotrophs with either PACAP, the cAMP analog 8-Br-cAMP, or the adenylate cyclase activator forskolin has been shown to activate the promoter of the glycoprotein hormone α-subunit gene in αT3-1 and LβT2 gonadotrophes [10,13], involving a tandem cAMP response element present in the glycoprotein hormone α-subunit promoter. The administration of the cAMP analog 8-Br-cAMP to JEG-3 human placental choriocarcinoma cells also upregulated glycoprotein hormone α-subunit gene promoter activity, but deletion and inactivation of the tandem CREs did not alter the 8-Br-cAMP responsiveness of the reporter gene [36]. These experiments were performed using transient transfection experiments of reporter plasmids to investigate the transcriptional responsiveness of the glycoprotein hormone α-subunit promoter. This method has the problem that transfected plasmids are not packed or are inefficiently packed into the chromatin environment of the nucleus and therefore exhibit incomplete nucleosomal organization [26,27]. Transfected plasmids exhibit a prokaryotic gene structure with a nonrestrictive transcriptional status that allows transcription factors and RNA polymerase to bind freely to DNA. In contrast, in eukaryotes, there is a restrictive ground state of the chromatin. In this study, we used recombinant lentiviruses to implant reporter genes into the chromatin of JEG-3 cells. This strategy ensured that the reporter genes were embedded into an ordered nucleosomal structure.

Schematic representation of integrated proviruses containing glycoprotein hormone α-subunit promoter/luciferase reporter genes. Both reporter genes contain human glycoprotein hormone α-subunit promoter sequence from −846 to +48. The αGSU.luc reporter gene contains the wild-type sequence of the human glycoprotein hormone α-subunit promoter, whereas a deletion of the tandem CRE was introduced in the αGSU.lucΔCRE reporter gene. The U3 region of the 5′ LTR is deleted. The proviruses contain additionally the woodchuck hepatitis virus posttranscriptional regulatory element (WPRE) and the HIV flap element.

**Figure 1:**
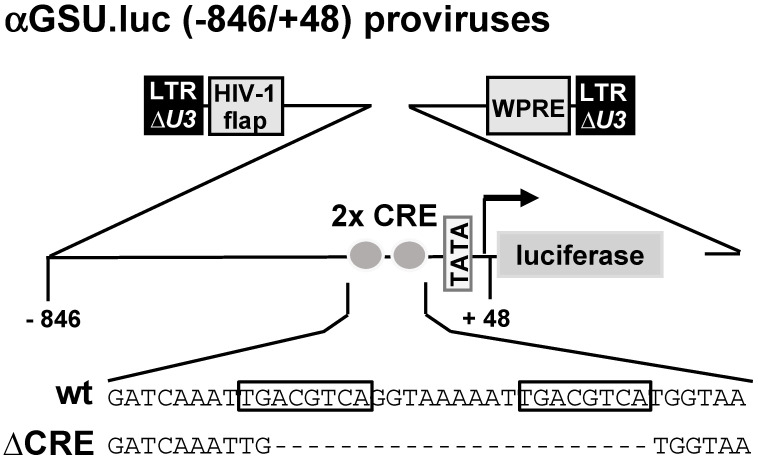
The glycoprotein hormone α-subunit promoter contains a tandem cAMP-responsive element (CRE).

Figure 1 shows schematically the integrated proviruses used in this study. The αGSU.luc reporter gene contains the human glycoprotein hormone α-subunit promoter (sequence −846 to +48) upstream of the luciferase open reading frame. The αGSU.lucΔCRE reporter gene contains a deletion of the tandem CRE within the promoter of the human glycoprotein hormone α-subunit gene.

To elucidate the molecular mechanism of stimulus-induced transcription of the glycoprotein hormone α-subunit gene, we chose JEG-3 cells, which have been used by many researchers in the past as a cellular model system for human trophoblasts, among others, to study the regulation of the glycoprotein hormone α-subunit gene. JEG-3 cells express human glycoprotein hormone chorionic gonadotropin (hCG) [30,31], i.e., the cells have the machinery to transcribe the glycoprotein hormone α-subunit gene. The cells also express the GnRH receptor [37], a Gαq-coupled receptor, so that it can be investigated whether stimulation of a Gαq-coupled receptor regulates transcription of the glycoprotein hormone α-subunit gene.

**Figure 2:**
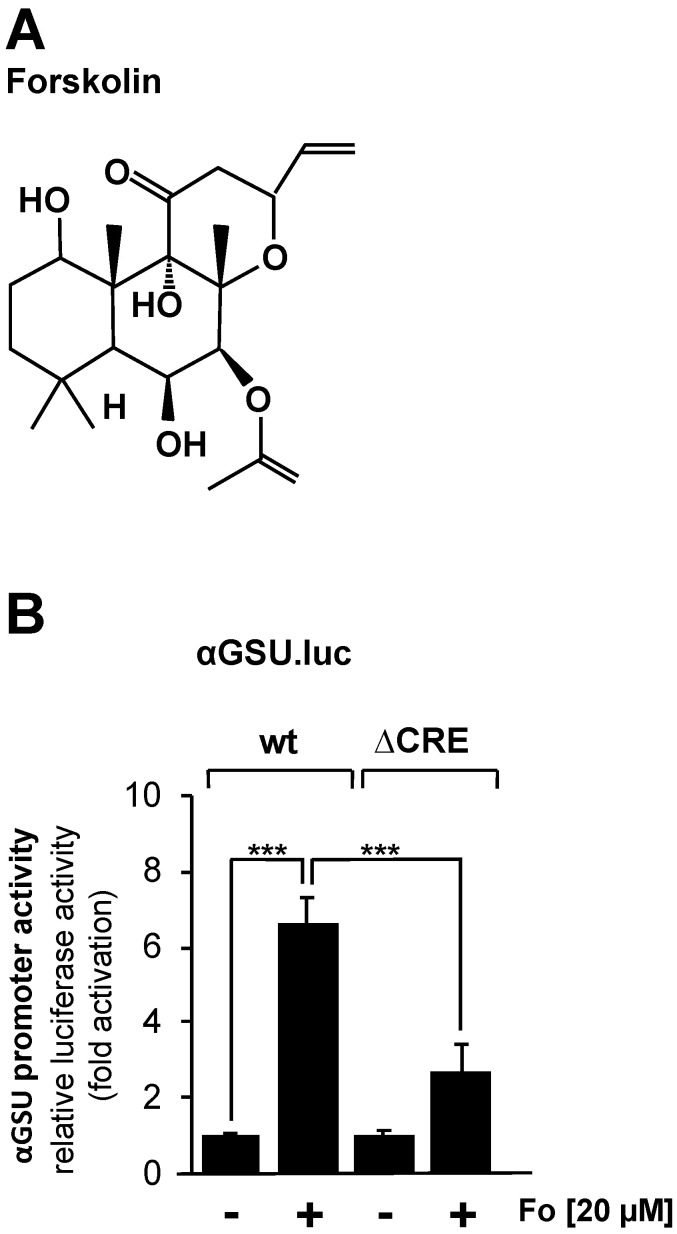
Stimulation of JEG-3 cells with forskolin increases the promoter activity of the human glycoprotein hormone α-subunit gene. (**A**) Chemical structure of forskolin. (**B**) JEG-3 cells were infected with a lentivirus containing the αGSU.luc or the αGSU.lucΔCRE reporter gene. Cells were maintained in serum-reduced medium containing 0.05% serum for 24 h and then stimulated with forskolin (20 μM) in serum-reduced medium for 24 h. Cell extracts were prepared, and luciferase activities and protein concentrations were determined. Luciferase activity was normalized to the protein concentration. Data shown are means +/− SD of five experiments performed in quadruplicate (*** *p* < 0.001).

JEG-3 cells were infected with a lentivirus containing either the αGSU.luc or the αGSU.lucΔCRE reporter gene, resulting in the integration of the reporter genes into the chromatin of JEG-3 cells. After infection, cells were incubated in serum-reduced medium for 24 h. Stimulation with forskolin (Figure 2A), an activator of adenylate cyclase, was performed in serum-reduced medium for 24 h to increase the intracellular cAMP concentration. Figure 2B shows that administration of forskolin to JEG-3 cells increased the activity of the glycoprotein hormone α-subunit promoter by 6.6-fold, whereas deletion of the tandem CREs resulted in a stimulation on the order of 2.7-fold. Thus, the glycoprotein hormone α-subunit promoter responded to increased cAMP levels in JEG-3 cells, and the tandem CRE is involved in the link between increased cAMP levels and enhanced transcription of the reporter gene controlled by the human glycoprotein hormone α-subunit promoter. However, deletion of the tandem CRE did not completely block the effect of forskolin on the human glycoprotein hormone α-subunit promoter.

### 3.2. Nuclear Expression of the Catalytic Subunit of cAMP-Dependent Protein Kinase in JEG-3 Cells Activates the Glycoprotein Hormone α-Subunit Promoter

**Figure 3:**
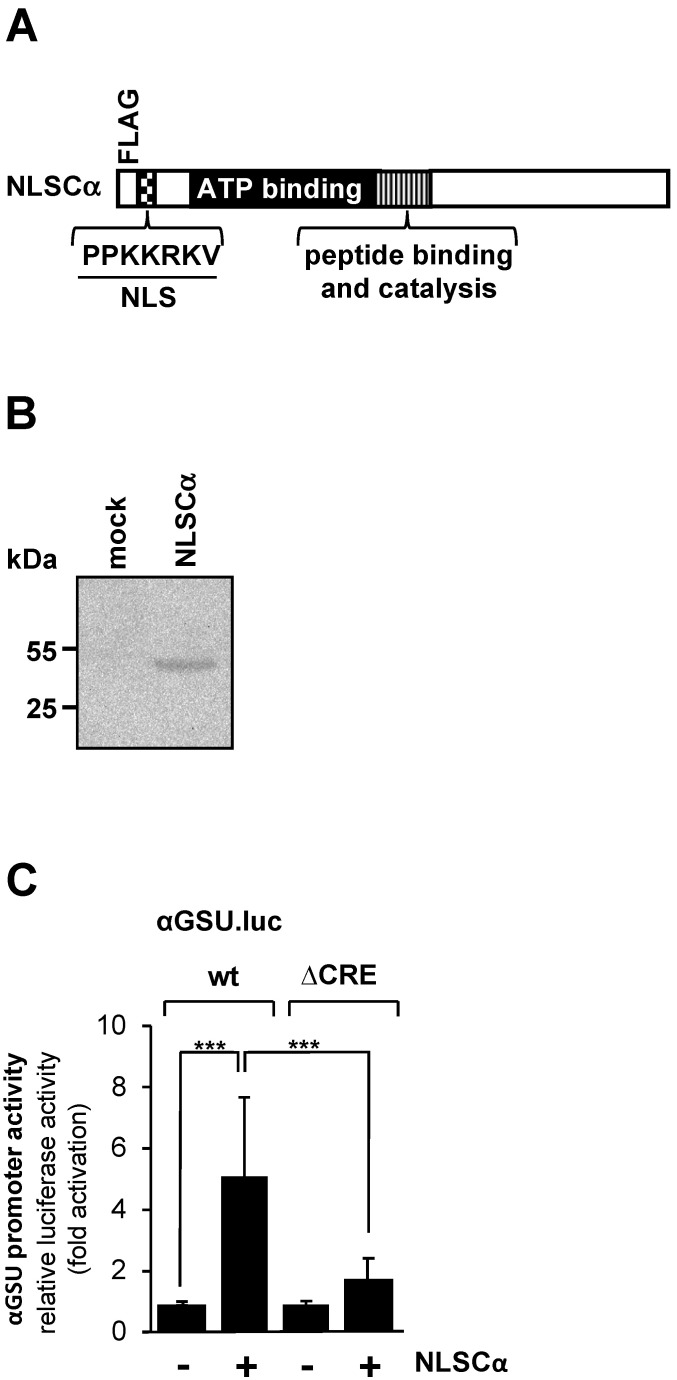
Expression of the catalytic subunit of cAMP-dependent protein kinase in JEG-3 cells leads to upregulation of the promoter activity of the human glycoprotein hormone α-subunit gene. (**A**) Modular structure of NLSCα. Shown are the catalytic center, ATP binding domain, NLS and FLAG epitope. (**B**) Expression of NLSCα in JEG-3 cells after infection of the cells with an NLSCα-encoding lentivirus. As a control, we analyzed JEG-3 cells infected with a lentivirus encoding β-galactosidase (mock). The Western blot was incubated with an antibody against the FLAG-tag. kDa, molecular-mass marker. (**C**) JEG-3 cells containing either the αGSU.luc or the αGSU.lucΔCRE reporter gene were infected with a lentivirus encoding either NLSCα or, as control, β-galactosidase, and incubated for 48 h in serum-starved medium. Cell extracts were prepared, and luciferase activities and protein concentrations were determined. Data shown are means +/− SD of three experiments performed in quadruplicate (*** *p* < 0.001).

Increased intracellular cAMP concentrations activate cAMP-dependent protein kinase (PKA) by binding to the regulatory domains of the holoenzyme and thus releasing the catalytic subunit. Other targets of cAMP are the exchange factors directly activated by cAMP (EPAC), which trigger an increase in intracellular Ca^2+^ [38]. We tested the effect of PKA on the promoter activity of the glycoprotein hormone α-subunit gene by expressing a modified version of the catalytic subunit of PKA (Figure 3A). The NLSCα mutant contains a nuclear localization signal (NLS) to support efficient translocation of the catalytic subunit into the nucleus. Translocation of the wild-type catalytic subunit into the nucleus is not very efficient and depends largely on diffusion [39]. NLSCα is expressed in JEG-3 cells following infection of the cells with an NLSCα-encoding lentivirus (Figure 3B). JEG-3 cells were infected with a lentivirus containing either the αGSU.luc or the αGSU.lucΔCRE reporter gene. In addition, cells were infected with a lentivirus encoding NLSCα or β-galactosidase as a control. Figure 3C shows that expression of NLSCα significantly stimulated the promoter activity of the glycoprotein hormone α-subunit gene in JEG-3 cells. Transcription of the αGSU.luc reporter was elevated 5.1-fold in the presence of NLSCα. Deletion of the tandem CREs significantly decreased the responsiveness of the glycoprotein hormone α-subunit promoter to NLSCα. Stimulation on the order of only 1.7-fold was measured (Figure 3C).

### 3.3. Expression of a Constitutively Active Mutant of MAP Kinase Kinase-6 in JEG-3 Cells Leads to Activation of the Glycoprotein Hormone α-Subunit Promoter

JEG-3 cells express TGF-β receptors type I and II that signal via activation of p38 MAP kinase [40], a proline-directed serine/threonine kinase involved in stress response, inflammation, and cancer [41]. p38 MAP kinase is also activated in αT3-1 gonadotrophs after stimulation of the cells with gonadotropin-releasing hormone (GnRH) [24]. p38 MAP kinase phosphorylates and activates the transcription factor ATF2, which is known to bind to the cAMP response element [33,42].

**Figure 4:**
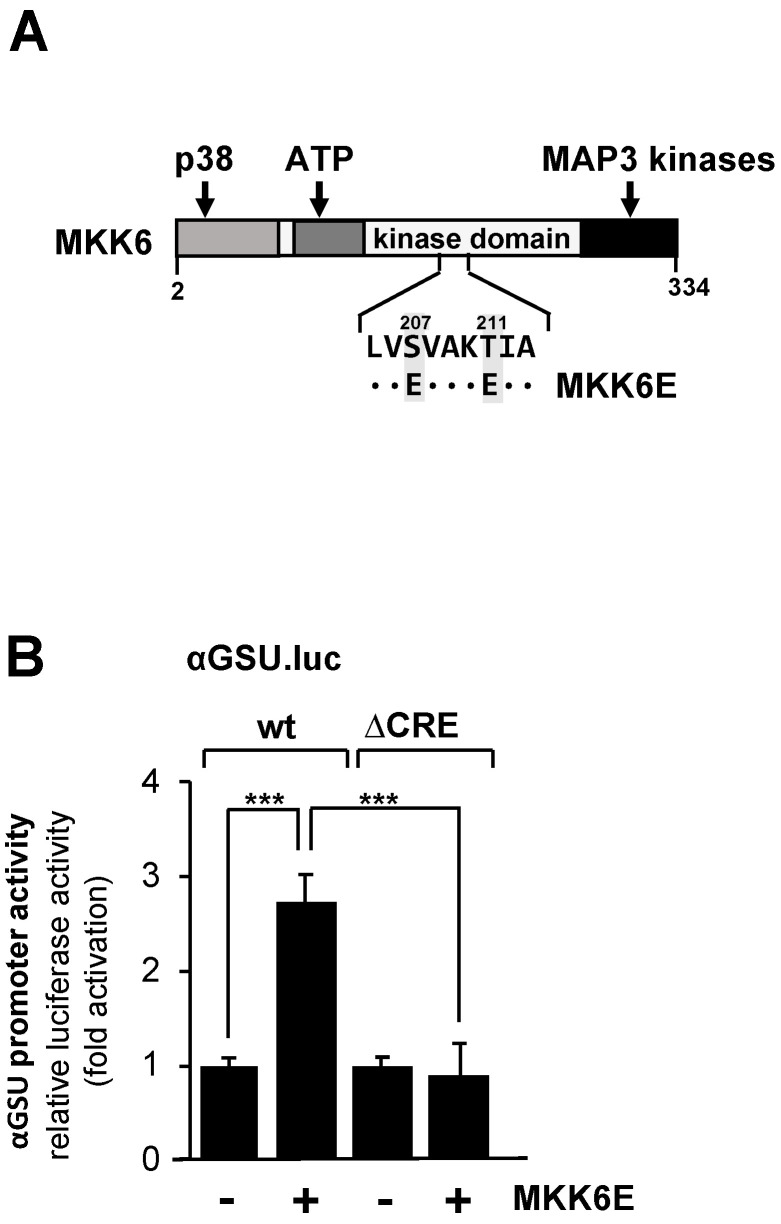
Expression of MKK6E in JEG-3 cells upregulates the promoter activity of the human glycoprotein hormone α-subunit gene involving the tandem CRE. (**A**) Modular structure of MKK6E, showing the C-terminal interaction site with MAP3 kinases, the ATP binding site, and the docking site for p38. The phosphorylation sites S207 and T211 were mutated to acidic glutamic acid residues. (**B**) JEG-3 cells were infected with a lentivirus containing either the αGSU.luc or the αGSU.lucΔCRE reporter gene. Cells were additionally infected with a lentivirus encoding either MKK6E or, as control, β-galactosidase, and incubated for 48 h later in serum-starved medium. Cell extracts were prepared, and luciferase activities and protein concentrations were determined. Data shown are means +/− SD of four experiments performed in quadruplicate (*** *p* < 0.001).

We therefore examined the effect of p38 MAP kinase activation on the promoter activity of the glycoprotein hormone α-subunit gene in JEG-3 cells by expressing a mutant of MAP kinase kinase-6, an upstream activator of p38 protein kinase. The MKK6E mutant contains two point mutations (S207E and T211E) that destroy the phosphoacceptor sites and introduce negative charges instead. MKK6E is constitutively active, and specificity towards p38 protein kinase has been demonstrated [43,44]. The modular structure of MKK6E is shown in Figure 4A.

JEG-3 cells carrying either a chromatin-embedded αGSU.luc or αGSU.lucΔCRE reporter gene were infected with a lentivirus encoding MKK6E or, as a control, β-galactosidase. Figure 4B shows that expression of MKK6E stimulated the promoter activity of the glycoprotein hormone α-subunit gene in JEG-3 cells by 2.7-fold, indicating that stimulation of the p38 protein kinase triggers expression of glycoprotein hormone α-subunit gene. In contrast, deletion of the tandem CRE completely abolished the responsiveness of the glycoprotein hormone α-subunit promoter to MKK6E (Figure 4B), indicating that the tandem CRE is the only genetic target sequence for p38 protein kinase signaling in the proximal promoter of the glycoprotein hormone α-subunit gene.

### 3.4. Expression of a Constitutively Active Form of Mitogen-Activated/Extracellular Signal Responsive Kinase Kinase Kinase-1 (MEKK1) Has Only Small Effect on the Promoter Activity of the Glycoprotein Hormone α-Subunit Gene in JEG-3 Cells

**Figure 5:**
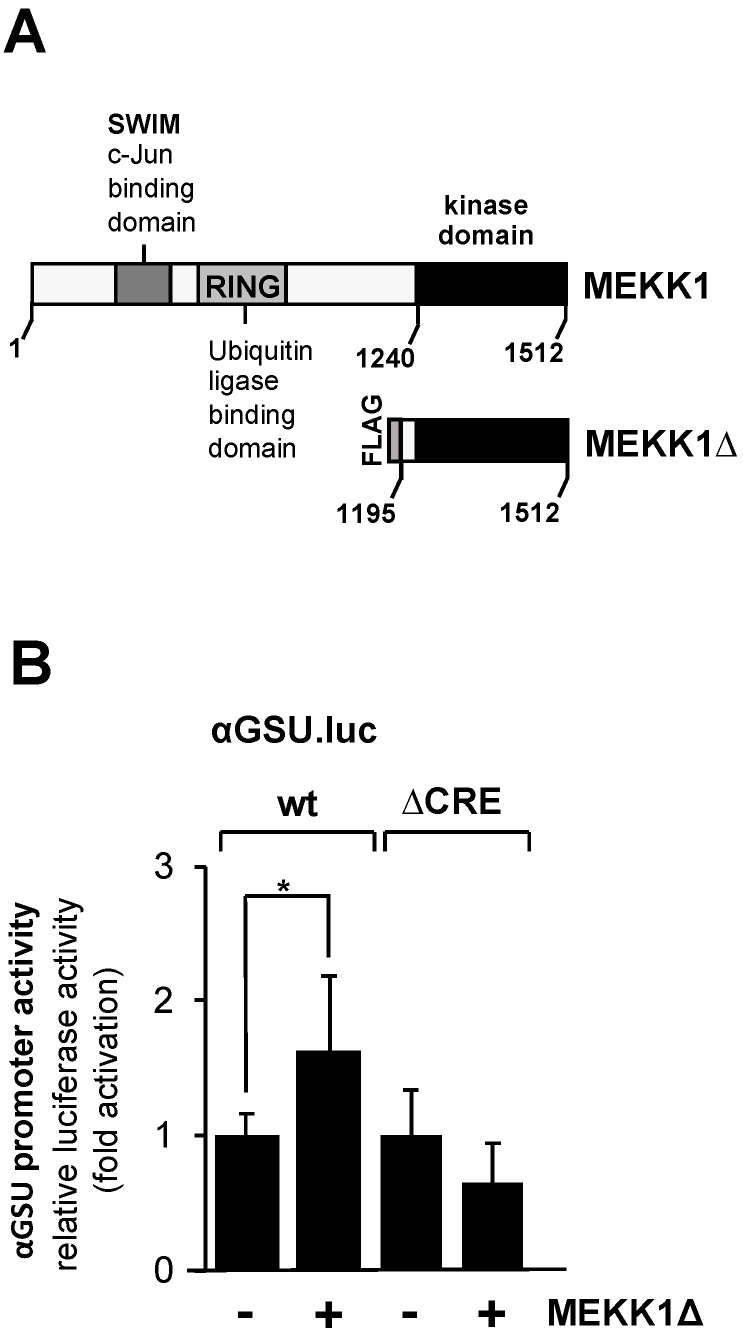
Expression of a truncated version of MEKK1 slightly stimulates the promoter of human glycoprotein hormone α-subunit gene in JEG-3 cells. (**A**) Modular structures of MEKK1 and MEKK1Δ showing the C-terminal catalytic domain of MEKK1 and multiple protein–protein interaction domains within the wild-type MEKK1 molecule. (**B**) JEG-3 cells were infected with recombinant lentiviruses which contained either the αGSU.luc or the αGSU.lucΔCRE reporter gene. In addition, we infected the cells with a lentivirus encoding either MEKK1Δ or β-galactosidase. After 48 h cells were harvested, cell extracts were prepared, and analyzed for luciferase activities and protein concentrations. Data shown are means +/− SD of three experiments performed in quadruplicate (* *p* < 0.05).

Based on in vitro DNA protein interaction studies, it has been suggested that the transcription factor c-Jun activates transcription of the glycoprotein hormone α-subunit gene via the tandem CRE in gonadotrophs but not in trophoblasts [25]. c-Jun is activated using phosphorylation involving JNK. We wanted to know whether activation of the JNK pathway stimulates the glycoprotein hormone α-subunit promoter. JNK was activated in JEG-3 cells by expressing a truncated version of MEKK1 with recombinant lentiviruses. The MEKK1 mutant designated as MEKK1Δ (Figure 5A) is constitutively active, and specificity for JNK activation has been demonstrated [43,45,46]. JEG-3 cells were infected with a lentivirus containing either the αGSU.luc or the αGSU.lucΔCRE reporter gene. In addition, cells were infected with a lentivirus encoding MEKK1Δ or β-galactosidase. Figure 5B shows that expression of MEKK1Δ only slightly stimulated the glycoprotein hormone α-subunit promoter in JEG-3 cells. Stimulation was on the order of 1.6-fold. Deletion of the tandem CREs completely abolished the responsiveness of the glycoprotein hormone α-subunit promoter to MEKK1Δ (Figure 5B). We conclude that activation of the JNK pathway has only a small if any effect on the expression of glycoprotein hormone α-subunit gene in JEG-3 cells.

### 3.5. Expression of an Activated Gαq-Coupled Designer Receptor in JEG-3 Cells Induces Glycoprotein Hormone α-Subunit Promoter-Controlled Gene Transcription

**Figure 6:**
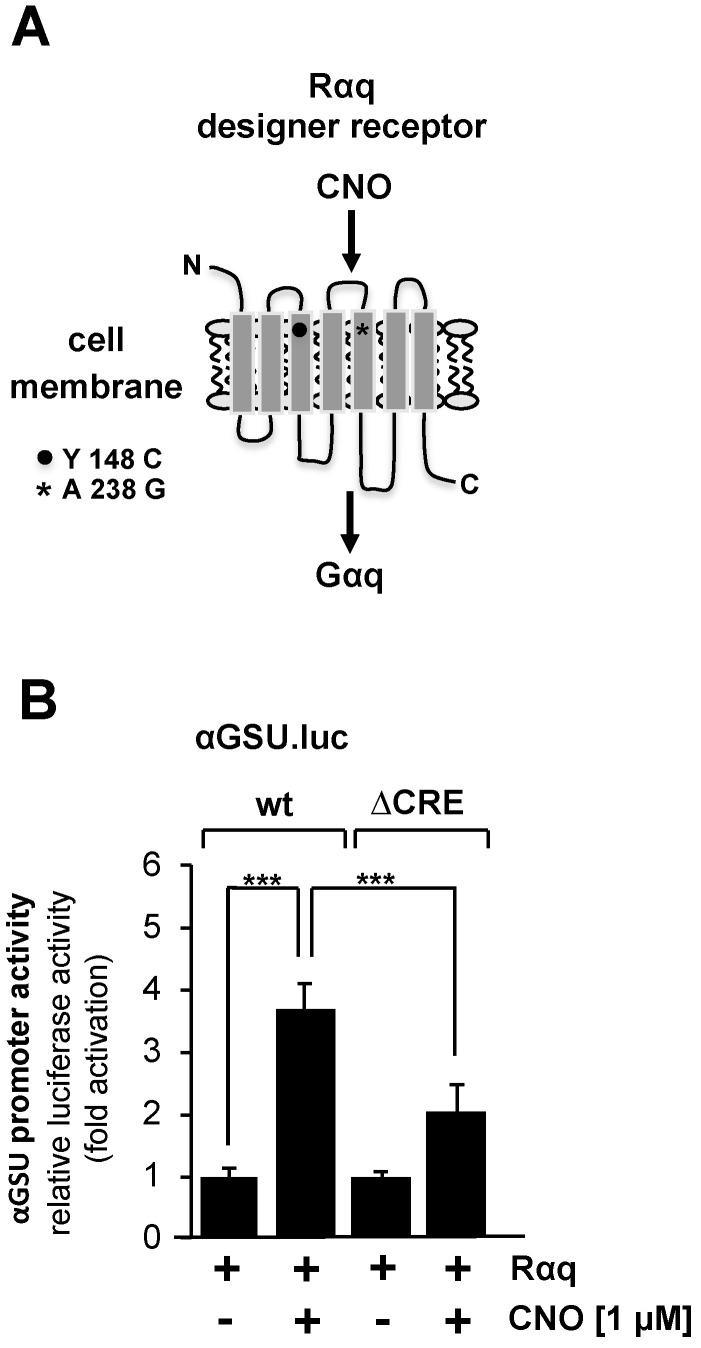
Stimulation of Gαq-coupled designer receptors in JEG-3 cells activates transcription of a chromatin-embedded reporter gene controlled by the promoter of the glycoprotein hormone α-subunit gene. (**A**) Modular structure of the Gαq-coupled designer receptor Rαq. (**B**) JEG-3 cells were infected with a lentivirus encoding the Gαq-coupled designer receptor Rαq. In addition, we infected the cells with a lentivirus containing either the αGSU.luc or the αGSU.lucΔCRE reporter gene. Cells were serum-starved for 24 h and then stimulated with CNO (1 μM) for 24 h in serum-reduced medium. Cell extracts were prepared, and luciferase activities and protein concentrations were determined. Data shown are means +/− SD of five experiments performed in quadruplicate (*** *p* < 0.001).

Stimulation of gonadotropin-releasing hormone (GnRH) receptors activates the glycoprotein hormone α-subunit promoter in αT3-1 gonadotrophs [9,10,15,16]. The GnRH receptor is one of the G protein-coupled receptors associated with hydrolysis of phosphatidylinositol 4,5-bisphosphate via Gαq and phospholipase Cβ. We specifically investigated the regulation of transcription of the glycoprotein hormone α-subunit gene by Gαq-coupled receptors in JEG-3 cells by expressing a Gαq-coupled designer receptor. This receptor is coupled to the G protein Gαq and is specifically activated by CNO [47,48] (Figure 6A).

Figure 6B shows that stimulation of the designer receptor with CNO resulted in an upregulation of reporter gene transcription controlled by the glycoprotein hormone α-subunit promoter on the order of 3.7-fold. The αGSU.lucΔCRE reporter gene containing deletion of the tandem CRE within the glycoprotein hormone α-subunit promoter was stimulated 2-fold in cells expressing an activated designer receptor (Figure 6B). We conclude that the activity of the glycoprotein hormone α-subunit promoter is regulated by stimulated Gαq-coupled designer receptors. However, deletion of the tandem CRE did not completely block the effect of receptor stimulation on human glycoprotein hormone α-subunit promoter activity.

### 3.6. Activation of the Glycoprotein Hormone a-Subunit Promoter in JEG-3 Cells Induced by Forskolin and the Gαq-Coupled Designer Receptor Depends on the Transcription Factor CREB

**Figure 7:**
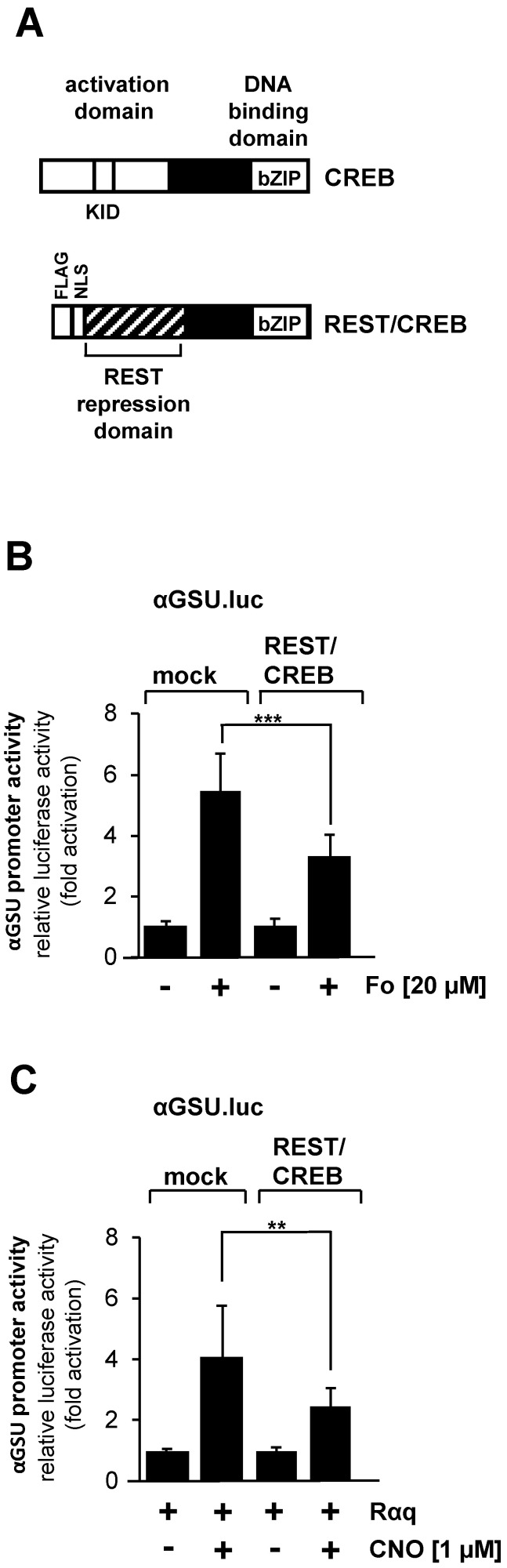
Activation of glycoprotein hormone α-subunit promoter by forskolin or stimulated designer receptor requires CREB. (**A**) Modular structure of CREB and the dominant-negative CREB mutant REST/CREB. Shown are the major functional domains of CREB, including the transcriptional activation domain, the kinase-inducible domain (KID and the basic-region/leucine zipper (bZIP) domain. The CREB mutant REST/CREB retains the bZIP domain, but lacks the activation domain. REST/CREB has an N-terminal transcriptional repression domain derived from the transcriptional repressor REST. (**B**) Expression of REST/CREB reduces the promoter activity of the glycoprotein hormone α-subunit gene after stimulation of JEG-3 cells with forskolin. Cells were infected with a lentivirus containing the αGSU.luc reporter gene. In addition, cells were infected with a lentivirus encoding either REST/CREB or, as control, β-galactosidase. Cells were serum-starved for 24 h and then stimulated with forskolin (20 μM) for 24 h in serum-reduced medium. Data shown are means +/− SD of three experiments performed in quadruplicate (*** *p* < 0.001). (**C**) Expression of REST/CREB reduces the promoter activity of the glycoprotein hormone α-subunit gene in JEG-3 cells after stimulation a Gαq-coupled designer receptor. Cells containing the αGSU.luc reporter gene were infected with a lentivirus encoding Rαq. In addition, cells were infected with a lentivirus encoding either REST/CREB or β-galactosidase. Cells were serum-starved for 24 h and then stimulated with CNO (1 μM) for 24 h in serum-reduced medium. Data shown are means +/− SD of three experiments performed in quadruplicate (** *p* < 0.01).

The tandem CRE within the promoter of the glycoprotein hormone α-subunit gene has been identified as a binding site for the transcription factor CREB [25]. CREB integrates numerous intracellular signaling cascades, particularly those leading to increased cAMP concentration in cells. In addition, CREB is activated after stimulation of Gαq-coupled designer receptors in HEK293 cells [28] or after stimulation of GnRH receptors in αT3-1 gonadotrophs [29]. We disrupted CREB function in JEG-3 cells by expressing a dominant-negative mutant of CREB, designated REST/CREB (Figure 7A), to dissect the involvement of CREB in stimulus-regulated activation of the glycoprotein hormone α-subunit promoter. REST/CREB binds to the CRE because it retaines the bZIP domain of CREB, which is used for dimerization and DNA binding. REST/CREB does not activate transcription due to a deletion of the activation domain of CREB. The repression domain of REST recruits histone deacetylases to the transcription unit, which place the chromatin-embedded reporter gene in a compacted state. We investigated the role of CREB in forskolin-stimulated JEG-3 cells. Cells were infected with a lentivirus containing the αGSU.luc reporter gene. In addition, we infected the cells with a lentivirus encoding either REST/CREB or, as control, β-galactosidase. Figure 7B shows that transcription of the αGSU.luc reporter gene was reduced by more than 40% in forskolin-stimulated JEG-3 cells in the presence of REST/CREB.

We next examined the effect of REST/CREB on designer receptor-mediated activation of the glycoprotein hormone α-subunit promoter. JEG-cells were infected with a lentivirus containing αGSU.luc. Cells were additionally infected with lentiviruses encoding the Gαq-coupled designer receptor Rαq, REST/CREB, or β-galactosidase. Cells were stimulated with CNO to activate the designer receptor. Figure 7C shows that gene transcription of the reporter gene controlled by glycoprotein hormone α-subunit promoter induced by stimulation of the designer receptor Rαq was significantly impaired in JEG-3 cells expressing REST/CREB. Transcription of the reporter gene was reduced by 45% in CNO-stimulated JEG-3 cells expressing the dominant-negative mutant of CREB. Thus, we conclude that activation of the glycoprotein hormone α-subunit promoter by either forskolin or following stimulation of a Gαq-coupled receptor is mediated by the transcription factor CREB.

## 4. Discussion

The gene encoding the glycoprotein hormone α-subunit is regulated by extracellular signaling molecules, and similarities and differences in the regulation of the α-subunit gene in gonadotrophs, thyreotrophs and placenta cells have been reported. In the present study, we investigated stimulus-specific regulation of the glycoprotein hormone α-subunit gene promoter using reporter genes that had been integrated into the genome of human placental JEG-3 choriocarcinoma cells using recombinant lentivirus technology. In this way, we ensured that the reporter gene was packed into a nucleosomal structure. This approach differs substantially from previous reports that based their conclusions on the results of transiently transfected plasmids.

The glycoprotein hormone α-subunit promoter contains a tandem cAMP response element (CRE), suggesting that elevated intracellular cAMP concentrations regulate α-subunit expression involving the CRE binding protein CREB. Increased cAMP concentrations have been shown to stimulate hCG expression in JEG-3 cells [49]. Our data showed that administration of forskolin to the cells, a compound that acts as an activator of adenylate cyclase, activated the promoter of the glycoprotein hormone α-subunit gene, confirming previously published data [20]. Expression of a dominant-negative mutant of CREB attenuated gene transcription of a reporter gene controlled by the glycoprotein hormone α-subunit promoter, suggesting that CREB likely mediates the effect of elevated cAMP concentrations on the glycoprotein hormone α-subunit gene. Deletion of the tandem CRE within the glycoprotein hormone α-subunit promoter significantly reduced transcription of the reporter gene, highlighting the importance of this genetic element in cAMP-induced gene transcription. It has been suggested that the gonadotroph-specific element within the glycoprotein hormone α-subunit promoter may additionally promote transcription as a result of elevated cAMP concentrations, explaining the fact that deletion of the tandem CRE did not completely blocked reporter gene transcription. Nonetheless, the tandem CRE is an important genetic landmark that converts increased cAMP concentration into a stimulation of glycoprotein hormone α-subunit gene transcription. This observation was confirmed in experiments showing that overexpression of the catalytic subunit of cAMP-dependent protein kinase in the nucleus led to a strong activation of the promoter of the glycoprotein hormone α-subunit gene.

Based on in vitro DNA-protein binding assays, it was suggested that ATF2 interacts with the promoter of the glycoprotein hormone α-subunit gene [25]. ATF2 is a basic region-leucine zipper transcription factor that can activate CRE-mediated gene transcription [33,42]. ATF2 is a substrate of p38 protein kinases, a stress-activated protein kinase. To activate ATF2, we expressed a constitutively active MAP kinase kinase-6 mutant in JEG-3 cells, which phosphorylates and activates p38 protein kinase. The results showed that the activity of the glycoprotein hormone α-subunit promoter was significantly increased, whereas a reporter gene under the control of the mutated glycoprotein hormone α-subunit promoter, lacking the tandem CRE, did not respond at all to MKK6E expression. We conclude that ATF2 is among the regulators of stimulus-induced activation of the glycoprotein hormone α-subunit gene. In this scenario, experimental evidence argues against the formation of a CREB-ATF2 heterodimer that binds to the glycoprotein hormone α-subunit promoter [33,50]. Expression of MKK6E in αT3-1 gonadotrophs did not result in activation of a reporter gene under the control of the glycoprotein hormone α-subunit promoter [24], suggesting that the p38-mediated signaling pathway has different significance in gonadotrophs and trophoblast cells. Expression of a truncated mutant of MEKK1, a potent activator of JNK and c-Jun, resulted in only minor activation of the glycoprotein hormone α-subunit promoter, suggesting that c-Jun is not a major player in stimulus-induced regulation of the glycoprotein hormone α-subunit gene. These results are consistent with the observation that JNK is not involved in the GnRH-induced activation of the glycoprotein hormone α-subunit promoter [14].

Many previous studies addressed the regulation of the promoter of the glycoprotein hormone α-subunit gene by GnRH. GnRH is essential for pituitary LH and FSH synthesis and secretion and for trophoblast cell hCG secretion. Disruption of GnRH signaling has been shown to reduce expression of the glycoprotein hormone α-subunit gene in the anterior pituitary and secretion of gonadotropic hormones [51]. Stimulation of GnRH receptors coupled to the G protein Gαq leads to hydrolysis of phosphatidylinositol 4,5-bisphosphate via activation of phospholipase Cβ. In this study we investigated the sensitivity of the glycoprotein hormone α-subunit promoter to stimulation of Gαq-couped receptors using chemogenetics technology.

Expression of a Gαq-coupled designer receptor in JEG-3 cells provided a non-redundant pair of receptor and ligand. The results of this study demonstrate that the glycoprotein hormone α-subunit promoter is responsive to activation of the Gαq-coupled receptor. Stimulation of Gαq-coupled receptors leads to an increase in intracellular Ca^2+^ following IP_3_-induced activation of the IP_3_ receptor, suggesting that an increase in the concentration of the second messengers Ca^2+^ is important for the regulation of the glycoprotein hormone α-subunit gene in the placenta.

The importance of the tandem CRE within the glycoprotein hormone α-subunit promoter to Gαq-coupled receptor stimulation has been a subject of debate [10,15]. Using chromatin integrated reporter genes, we demonstrated in this study that deletion of the tandem CRE within the promoter of the glycoprotein hormone α-subunit gene significantly reduced reporter gene transcription, suggesting that the tandem CRE is a target of Gαq-coupled receptor signaling. Based on the fact that CREB is activated upon stimulation of Gαq-coupled receptors [28,29], we performed expression experiments with a dominant-negative mutant of CREB and showed that expression of this mutant attenuated transcription regulated by the promoter of the glycoprotein hormone α-subunit gene. Thus, CREB is likely the final regulator of the signaling cascade linking Gαq-coupled receptors and the glycoprotein hormone α-subunit gene.

Stimulation of Gαq-coupled receptors has been shown to activate several MAP kinases, including p38 protein kinase [14,24,52]. Moreover, stimulation of the designer receptor leads to an increase in the transcriptional activation potential of ATF2 in JEG-3 placental cells (G. Thiel, unpublished observations). We therefore propose that ATF2, in addition to CREB, regulates the expression of the glycoprotein hormone α-subunit gene in JEG-3 choriocarcinoma cells after stimulation of Gαq-coupled receptors.

## 5. Conclusions

**Figure 8:**
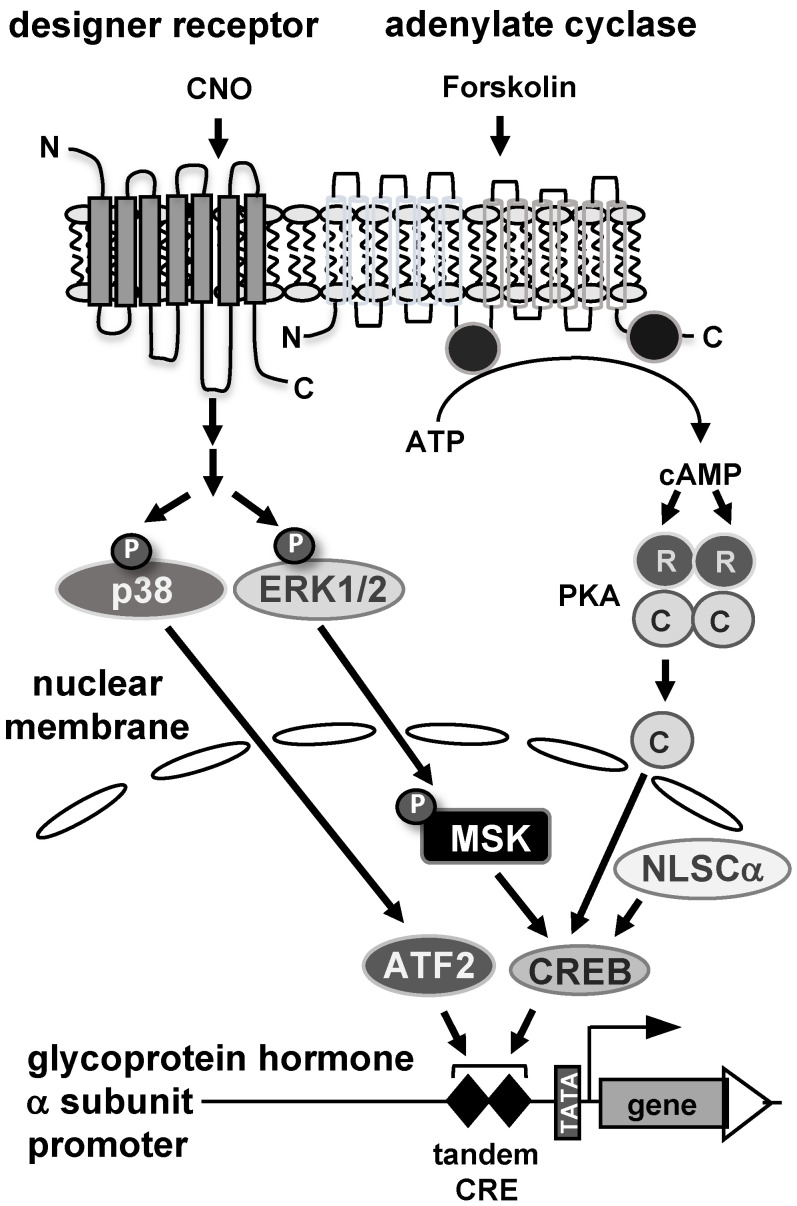
Stimulus-induced activation of the glycoprotein hormone α-subunit promoter in JEG-3 placental choriocarcinoma cells. Stimulation of adenylate cyclase by forskolin triggers the biosynthesis of cAMP, which in turn binds to the regulatory subunit of cAMP-dependent protein kinase (PKA), releasing the catalytic subunit. The catalytic subunit translocates to the nucleus and activates the glycoprotein hormone α-subunit promoter. A similar effect is seen after nuclear expression of a catalytic subunit mutant. Stimulation of Gαq-coupled designer receptors with the designer drug CNO activates stimulus-responsive protein kinases in JEG-3 cells, including extracellular signal-regulated protein kinase (ERK 1/2) and p38 protein kinase. Phosphorylated and activated ERK1/2 translocates to the nucleus and phosphorylates mitogen and stress-induced protein kinase (MSK), which in turn phosphorylates and activates die transcription factor CREB. In addition, stimulation of Gαq-coupled designer receptors leads to activation of p38 protein kinase, which phosphorylates and activates the transcription factor ATF2. CREB and ATF2 function as nuclear regulators of glycoprotein hormone α-subunit gene transcription.

In this study, stimulus-specific regulation of the glycoprotein hormone α-subunit gene promoter was investigated using reporter genes integrated into the genome of human JEG-3 placental choriocarcinoma cells. Figure 8 summarizes the results. The promoter of the glycoprotein hormone α-subunit gene responds to elevated cAMP concentrations, activation of protein kinases PKA and p38, and stimulation of Gαq-coupled receptors. An important role of the cAMP signaling pathway in trophoblast cells to regulate glycoprotein hormone α-subunit gene transcription has been proposed. This study using a chromatin-integrated reporter gene containing a deletion of the tandem CRE within the glycoprotein hormone α-subunit promoter demonstrated the importance of this genetic element in stimulus–transcription coupling after increasing the intracellular cAMP concentration with forskolin. Moreover, we showed for the first time that expression of the catalytic subunit of cAMP-dependent protein kinase in the nucleus increased the activity of the α-subunit promoter. This study showed, in addition to the cAMP signaling pathway, that stress-induced signals (via activation of p38 protein kinase) and hormonal signaling (via stimulation of Gαq-coupled receptors) also activate the expression of the glycoprotein hormone α-subunit. Both the stress-induced and the hormonally induced signaling cascades converge to the tandem CRE within the glycoprotein hormone α-subunit promoter and utilizes the transcription factor CREB and ATF2 as nuclear regulators of the intracellular signaling cascades that induce transcription of the glycoprotein hormone α-subunit gene. The tandem CRE within the glycoprotein hormone α-subunit promoter therefore acts as a convergence point for multiple signaling cascades.

## Data Availability

Data are contained within the article.
